# Learning Fine-Grained Video Anomaly Detection from Normal Videos

**DOI:** 10.3390/s26134314

**Published:** 2026-07-07

**Authors:** Ruqin Wang, Yasumasa Tamura, Masahito Yamamoto

**Affiliations:** 1 Graduate School of Information Science and Technology, Hokkaido University, Sapporo 001-0021, Japan; 2 Faculty of Information Science and Technology, Hokkaido University, Sapporo 060-0814, Japan; ytamura@ist.hokudai.ac.jp (Y.T.); masahito@ist.hokudai.ac.jp (M.Y.)

**Keywords:** video anomaly detection, weak supervision, vision–language model

## Abstract

Video anomaly detection (VAD) aims to identify abnormal events in videos. Due to the lack of high-quality training data with detailed annotations, current VAD methods can only produce video-level predictions. To remedy this, several methods attempt to synthesize pseudo video anomalies. However, these methods suffer from low realism and coarse annotations, which limits their performance in real-world scenarios. In this paper, we propose a framework for unsupervised anomaly video generation from solely normal videos, leveraging VLMs to generate structured textual descriptions of anomalies conditioned on the perception of this video. Then, abnormal segments are synthesized using VLMs based on the synthetic textual descriptions. As our framework is highly controllable, video-level and region-level labels can be obtained to provide fine-grained annotations. On top of the synthetic data, we develop a fine-grained VAD network to simultaneously produce video-level, frame-level, and region-level predictions. Experiments show that our method achieves remarkable fine-grained VAD performance.

## 1. Introduction

Video anomaly detection (VAD) aims at detecting abnormal events within video sequences [1], playing a critical role in real-world applications such as safety monitoring and industrial inspection [2,3]. During the past decades, extensive research has been conducted, achieving promising progress. Due to the high labor cost to annotate frame-level and region-level anomalies, most existing studies formulate VAD as a video-level classification task; that is, models are trained to determine whether an untrimmed video contains any anomalies, without explicitly learning when or where they occur. Representative works include STEAD [4], future frame prediction [5], and spot-anomaly [6], which focus on weakly supervised learning to mitigate the lack of dense labels.

**Figure 1:**
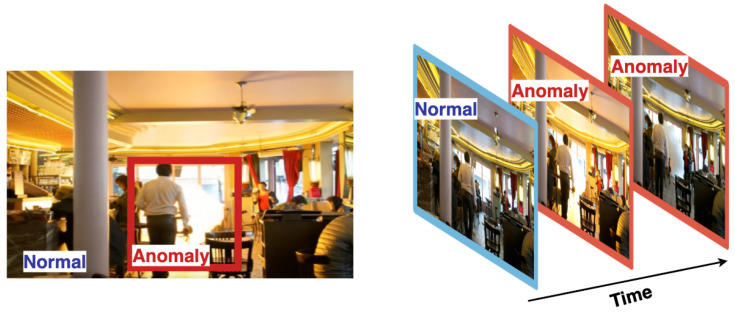
Fine-grained annotations for abnormal events at both the frame and region level, illustrating the necessity of precise spatial-temporal labeling in practical VAD applications.

While previous weakly supervised paradigms produced promising results on benchmark datasets, they suffer a fundamental limitation in that the detection results are too coarse to meet the requirements of downstream applications like automatic fire suppression. More specifically, video-level predictions produced by existing methods only indicate the presence of anomalies without providing their spatiotemporal location in the video sequence. As a result, frame-wise manual identification is usually required to further obtain fine-grained details, as shown in Figure 1.

To bridge this gap, we focus on enabling fine-grained video anomaly detection by addressing its fundamental limitation: the lack of training data with detailed annotations. While most existing datasets provide only video-level labels, we propose an unsupervised anomaly video generation framework that is capable of synthesizing videos containing diverse anomalous events with detailed annotations. Our framework is prompt-driven and flexibly controllable, allowing frame-level and region-level annotations to be obtained alongside video-level labels. On top of our synthetic data, we further develop a simple yet powerful baseline with multiple heads to achieve fine-grained video anomaly detection.

Our main contributions are summarized as follows:

- We propose an unsupervised anomaly video generation pipeline that first captures semantic descriptions from normal frames, then infers plausible anomalous events and their spatial locations, and finally synthesizes anomalous video segments.
- The proposed pipeline is able to produce video-, frame-, and region-level annotations, providing a scalable source of fine-grained supervision without requiring manual annotation.
- We develop a fine-grained VAD baseline on top of our synthetic data which is able to produce frame-level and region-level detection in addition to video-level labels.
- We conduct experiments on existing VAD methods by incorporating the synthetic data for training. The improved performance demonstrates the effectiveness of the synthetic data.

## 2. Related Work

In this section, we first briefly review recent video anomaly detection methods with a focus on weakly supervised approaches and anomaly synthesis approaches. Then, we discuss recent advances in general video generation.

### 2.1. Video Anomaly Detection

The objective of video anomaly detection (VAD) is to identify unusual or dangerous events in untrimmed videos [7]. Due to the high labor cost of obtaining dense spatial-temporal annotations, most existing methods formulate VAD as a weakly supervised learning task using only video-level labels [8]. To achieve competitive performance under weak supervision, many approaches adopt multiple instance learning (MIL) frameworks [8,9]. Recent works further improve localization and detection accuracy by incorporating temporal consistency [10] and attention-based refinement [6]. Despite notable improvements, the performance of weakly supervised VAD models is still constrained by the limited scale and diversity of existing datasets. To remedy this, benchmarks such as UCF-Crime [8], XD-Violence [9], and STEAD [4] provide long-form videos with sparse annotations. Recent benchmarks such as CUVA [11] further highlight this challenge by offering broader coverage of anomaly categories. The anomaly diversity remains limited, as real-world anomalies are rare and hard to collect.

To compensate for data scarcity, various anomaly synthesis techniques have been explored. Early efforts employ noise injection [12] to produce anomalous videos. More advanced approaches synthesize anomalous segments via splicing normal and abnormal clips [13] or simulation [14]. However, these methods frequently suffer artifacts at the boundary of objects. To improve synthesis quality and scalability, SVTA [15] leverages text prompts to guide anomaly generation, while C2FPL [16] applies a coarse-to-fine pseudo-labeling strategy to boost detection performance. Nevertheless, most existing generation pipelines rely on predefined anomaly types or templates and fail to cover diverse real-world anomalies.

### 2.2. General Video Generation

The field of video generation has witnessed rapid progress driven by foundation models. While early attempts focused on generating short and low-resolution clips from noise or motion priors, recent advances integrate LLMs and VLMs to support text-conditioned, scene-consistent, and high-resolution video generation [17,18], making significant advances in terms of controllability, fidelity, and temporal coherence. State-of-the-art approaches typically fall into three categories: autoregressive generation [19], diffusion-based methods [20,21], and transformer-based architectures [22,23]. These models generate videos from textual prompts, semantic layouts, or motion cues, allowing for more flexible and context-aware synthesis. Layout-guided frameworks [23,24] further enhance spatial consistency by conditioning generation on segmentation masks or bounding boxes, while vision–language pretraining allows for stronger alignment between textual and visual content [17,18].

## 3. Methodology

Despite recent advances in video generation, synthesizing anomalous videos with fine-grained spatiotemporal annotations remains under-explored. We address this limitation by proposing an unsupervised anomaly video generation pipeline based on VLMs, which provides scalable fine-grained supervision from solely normal videos.

### 3.1. Overview

**Figure 2:**
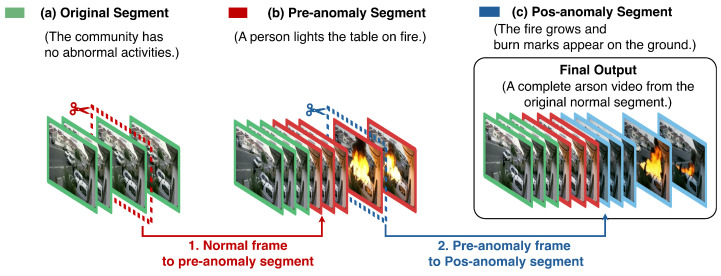
Overview of our proposed framework. Our pipeline first uses an LLM to perceive the normal video and generate spatial-temporal descriptions of abnormal events to guide the video generation process. This produces realistic anomaly videos with automatic fine-grained labels, enabling the training of detectors that extend beyond video-level prediction.

In this paper, we focus on a fine-grained anomaly detection setting, where the model is required to simultaneously predict video-level anomalies ${\hat{y}}^{\mathrm{video}}$, frame-level scores ${\hat{y}}^{\mathrm{frame}}$, and region-level bounding boxes ${\hat{y}}^{\mathrm{region}}$. To achieve this, the objective of our framework is to synthesize video anomalies with fine-grained annotations. As illustrated in Figure 2, our framework consists of three stages. Given a normal video segment $D_{r}$, we randomly select a frame *I* and then generate an abnormal video segment ${\hat{V}}_{v}^{\mathrm{abn}}\in D_{v}$ conditioned on it. With an elaborate video synthesis framework, the synthetic abnormal video segment is controllable such that frame-level and region-level annotations can be obtained.

### 3.2. Anomaly Generation Framework

**Figure 3:**
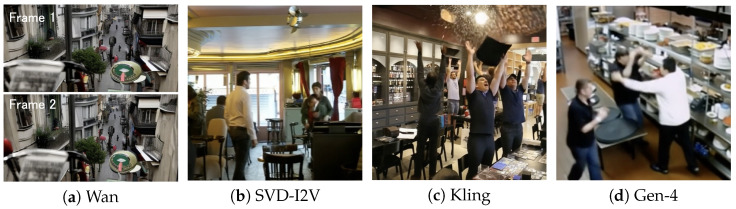
Representative results generated by different video generation models using the same prompt. Wan suffered from object distortion, SVD-I2V introduced noticeable artifacts, and Kling exhibited unstable multi-person dynamics. In contrast, Gen-4 Turbo achieved higher realism while preserving a stable surveillance camera style without abrupt changes in sharpness or color.

In preliminary experiments, we compared several text generation models (DeepSeek, Qwen, and GPT-4o) and multiple video generation models (Wan, Kling, Sora, Gen-4 Turbo, SVD-I2V, and SVDXT). Representative examples are shown in Figure 3. Among these candidates, GPT-4o and Gen-4 Turbo demonstrated superior semantic consistency and visual quality. Therefore, without loss of generality, our framework employs GPT-4o and Gen-4 Turbo for abnormal video synthesis.

**Figure 4:**
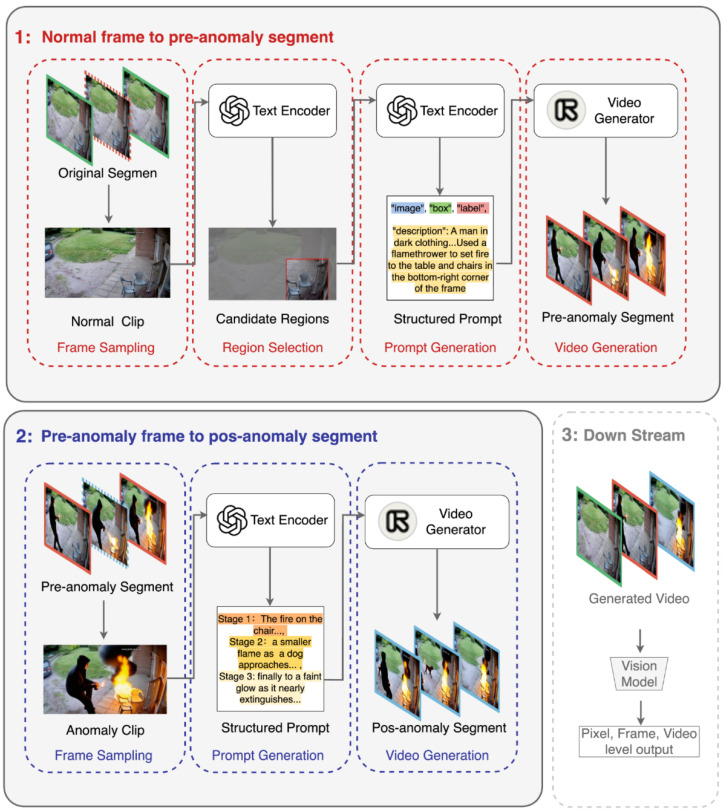
An illustration of our pipeline that synthesizes a pre-anomaly segment that smoothly transitions from normal frames toward the upcoming event.

Since abnormal events do not emerge or disappear instantaneously, we divided each synthesized abnormal segment into two stages: a pre-anomaly phase, during which the event gradually developed, and a post-anomaly phase, during which the abnormality progressively subsided. Based on this observation, the proposed synthesis pipeline consists of three key steps:

Step 1: Normal Frame to Pre-Anomaly Segment. As illustrated in Figure 4, given a normal video, a frame is randomly selected, and GPT-4o is employed to infer semantically meaningful regions (e.g., a table, a sidewalk, or an entrance) where abnormal events are likely to occur based on anomaly types and spatial configurations. Next, as shown in Algorithm 1, GPT-4o generates a structured prompt that describes a plausible pre-anomaly scene. The prompt consists of four components: image, box, label, and description. Afterward, this prompt is passed to Gen-4 Turbo to synthesize a pre-anomaly segment that introduces early signs of the anomaly in a spatially and temporally consistent manner.

Step 2: Pre-Anomaly to Post-Anomaly Segment. To enable a smooth transition from abnormal to normal frames, as shown in Algorithm 2 the synthetic pre-anomaly segment and its context are fed to GPT-4o to produce a multi-stage description that captures the temporal evolution of the scene. Then, the resultant description is passed to Gen-4 Turbo to synthesize a corresponding post-anomaly segment for smooth transition to normal frames.

Step 3: Video Selection for Fine-Grained Detection. By combining the original normal segment with generate pre-anomaly and post-anomaly segments, a full synthetic video is obtained. Afterward, we sample 16 representative frames within each video and feed them with the original description into GPT-4o to identify whether the generated videos match the corresponding prompts. Finally, the videos with high matching scores are selected for the downstream fine-grained VAD task.
**Algorithm 1** Pre-anomaly segment generation**Require:** Normal video segment $V=\{f_{1},f_{2},\ldots ,f_{T}\}$, anomaly type *a*, text encoder GPT-4o, video generator Gen-4 Turbo**Ensure:** Pre-anomaly video segment $V_{\mathrm{pre}}$  1: Randomly sample a frame $f_{k}$ from V  2: Use GPT-4o to infer semantically meaningful candidate anomaly regions:        $R=\{r_{i}\}$  3: Select region $r^{*}\in R$ according to anomaly type *a*  4: Construct a structured prompt $P_{\mathrm{pre}}$ consisting of an image $f_{k}$, bounding box of $r^{*}$, label *a*, and textual description  5: Generate pre-anomaly segment:        $V_{\mathrm{pre}}\leftarrow \text{Gen-4}\;\mathrm{Turbo}\left(P_{\mathrm{pre}}\right)$         **return** $V_{\mathrm{pre}}$

**Algorithm 2** Post-anomaly segment generation
**Require:** Pre-anomaly segment $V_{\mathrm{pre}}$, text encoder GPT-4o, video generator Gen-4 Turbo**Ensure:** Post-anomaly video segment $V_{\mathrm{post}}$  1: Sample representative frames from $V_{\mathrm{pre}}$:          $F_{\mathrm{pre}}=\left\{f_{\mathrm{pre}}^{1},\ldots ,f_{\mathrm{pre}}^{m}\right\}$  2: Use GPT-4o to generate a multi-stage temporal description          $D=\{d_{1},d_{2},\ldots ,d_{n}\}$  3: Generate post-anomaly segment:          $V_{\mathrm{post}}\leftarrow \text{Gen-4}\;\mathrm{Turbo}\left(D\right)$          **return** $V_{\mathrm{post}}$


### 3.3. FG-VAD Network

**Figure 5:**
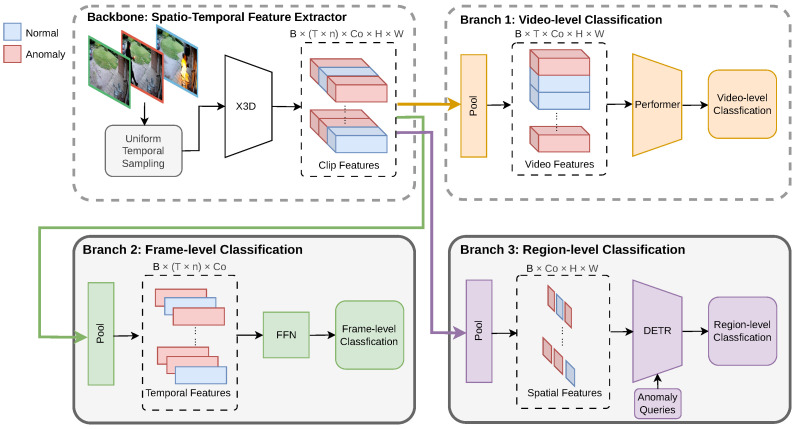
FG-VAD network architecture. Based on a standard video-level classification pipeline, we extract features using a shared X3D backbone and introduce two additional heads for frame-level and region-level prediction, forming a multi-branch structure. These two heads enable the model to tackle new tasks of temporal localization and spatial anomaly detection, in addition to the original video-level classification.

On top of our synthetic data rich in fine-grained annotations, we developed a fine-grained video anomaly detection network. The architecture of our proposed network is shown in Figure 5, which consists of a shared spatiotemporal feature extractor and three task-specific branches.

Spatio-Temporal Feature Extractor. We first divide the input video into *n* clips, each containing *T* frames, with a predefined sampling rate *r* that selects one frame every *r* frames. The resultant clip sequence is denoted as ${\left\{V_{i}\in R^{B\times T\times H\times W\times 3}\right\}}_{i=1}^{n}$. Next, each clip is encoded using a shared X3D backbone to produce spatiotemporal features $F_{\mathrm{clip}}^{i}\in R^{B\times T\times C\times H\times W}$. The features of all *n* clips are concatenated along the temporal dimension to form a unified video-level representation $F_{\mathrm{video}}\in R^{B\times (T\times n)\times C\times H\times W}$, which is then fed to subsequent parallel branches.

Video-Level Branch. This branch performs anomaly classification over the entire video. We apply spatial average pooling to $F_{\mathrm{video}}$ to obtain $F_{\mathrm{seq}}\in R^{B\times (T\times n)\times C}$. Then, $F_{\mathrm{seq}}$ is fed into the Performer encoder [25] to capture long-range temporal dependencies and produce ${\hat{y}}^{\mathrm{video}}\in R^{B\times K}$, where *K* is the number of video-level categories (typically K=2, i.e., normal or abnormal). Only videos classified as abnormal are passed to the frame-level branch for finer analysis.

Frame-Level Branch. This branch performs temporal localization of anomalies within each video. Specifically, $F_{\mathrm{video}}$ is fed to a spatial pooling layer to obtain $F_{\mathrm{frame}}\in R^{B\times (T\times n)\times C}$. Then, the feature of each frame is passed through a feedforward network (FFN) to estimate its anomaly probability ${\hat{y}}^{\mathrm{frame}}\in R^{B\times (T\times n)}$. Only frames with anomaly scores above a certain threshold are forwarded to the region-level branch for spatial analysis.

Region-Level Branch. This branch localizes anomalous regions within individual frames. For each abnormal frame, we apply temporal average pooling to the corresponding clip features to obtain $F_{\mathrm{region}}\in R^{B\times C\times H\times W}$. Then, $F_{\mathrm{region}}$ is fed into a transformer-based decoder along with $N_{q}$ learnable anomaly queries. The model predicts a set of bounding boxes:

$${\hat{y}}^{\mathrm{region}}={\left\{\left(x_{i},y_{i},w_{i},h_{i}\right)\right\}}_{i=1}^{N_{q}},\;{\hat{y}}^{\mathrm{region}}\in R^{N_{q}\times 4},$$

where $\left(x_{i},y_{i}\right)$ denotes the center coordinates and $\left(w_{i},h_{i}\right)$ represent the width and height of the predicted anomalous region, respectively. In our implementation, we set $N_{q}=1$, meaning only one bounding box is predicted per frame, to focus on the most salient anomaly. This simplified setting follows existing weakly supervised VAD datasets and may not fully handle complex scenarios involving multiple simultaneous anomalies.

### 3.4. Loss Function

To enable effective learning at different levels of anomaly granularity, the total loss used for joint optimization is defined as follows:

$$L_{\mathrm{total}}=L_{\mathrm{video}}+L_{\mathrm{frame}}+L_{\mathrm{region}}+\lambda_{1}\cdot L_{\mathrm{regularization}}.$$

Here, the regularization term encourages temporal consistency across adjacent frames by normalizing frame-wise feature magnitudes, following the temporal smoothness regularization used in [10]. This stabilizes training and prevents the model from overfitting to noisy frame-level fluctuations. For simplicity, equal weights are assigned to the video-level, frame-level, and region-level losses.

Video-Level Loss. The video-level loss encourages the model to assign higher anomaly scores to abnormal clips while minimizing intra-class variance. Let $s_{n}$ and $s_{a}$ be the scores for normal and abnormal inputs. The video-level loss $L_{\mathrm{video}}$ is defined as follows:

$$L_{\mathrm{video}}=L_{\mathrm{ranking}}\left(s_{n},s_{a}\right).$$

Frame-Level Loss. To learn the location of temporal anomalies, we introduce a frame-wise classification head. Given a sequence of temporal features ${\left\{f_{t}\right\}}_{t=1}^{T}$ and the corresponding binary labels ${\left\{y_{t}\right\}}_{t=1}^{T}$, we apply a binary cross-entropy (BCE) loss at each timestep:

$$L_{\mathrm{frame}}=\frac{1}{T}\sum_{t=1}^{T}\mathrm{BCE}\left({\hat{y}}_{t},y_{t}\right),$$

where ${\hat{y}}_{t}$ is the predicted anomaly score for frame *t*.

Region-Level Loss. During training, the decoder output $\hat{B}$ is compared with the ground truth bounding box *B* using a set prediction loss:

$$L_{\mathrm{region}}=L_{\mathrm{box\_}\_\mathrm{reg}}\left(\hat{B},B\right)+\lambda_{2}\cdot L_{\mathrm{IoU}}\left(\hat{B},B\right),$$

where the first term is an $\ell_{1}$ regression loss on box coordinates and the second term is a generalized IoU loss [26].

## 4. Experiments

### 4.1. Datasets

To highlight the limitations of existing datasets and motivate our proposed benchmark, we compared representative VAD datasets across four criteria in Table 1: (1) whether annotations could be automatically generated, (2) whether fine-grained labels were provided, (3) dataset size, and (4) scene diversity, which are further discussed in the Supplementary Materials. As shown in Table 2, most existing datasets only provide video-level or sparse temporal labels, lacking dense spatial annotations necessary for fine-grained VAD. To overcome these limitations, we introduce a scalable synthetic dataset that produces diverse anomaly videos with controllable frame- and region-level annotations. Detailed statistics are provided in Table 3. Although some categories cannot yet be synthesized, the additional synthetic data for other classes helps reduce inter-class ambiguity and improves the overall decision boundary, leading to consistent gains even on unsynthesizable categories.

### 4.2. Implementation Details

During training, videos were sampled at 30 FPS and resized to 320×320. Three backbones (C3D [30], I3D [31], and X3D [32]) were employed following their respective standard implementations. The AdamW optimizer was employed to optimize the trainble parameters for 50 epochs. More architectural and optimization details are provided in the Supplementary Materials.

### 4.3. Performance Evaluation

To evaluate the effectiveness of our method, we compared it with several state-of-the-art video anomaly detection approaches, including both weakly supervised and fine-grained localization models. All models were trained and evaluated on the same test set from UCF-Crime. For our method, our synthetic dataset was used as a complementary training set.

Video-Level Prediction. We evaluated video-level performance to assess the effectiveness of the proposed synthetic data across multiple representative backbone architectures. Note that the network architectures were kept unchanged, with only the training data being augmented by our synthetic samples. As shown in Table 4, incorporating the proposed synthetic data generally led to improved performance across different backbone architectures, suggesting that the generated data provided useful supervision for video anomaly detection.

As shown in Table 5, almost all anomaly categories benefited from the proposed synthetic data. Particularly, sudden large-area anomalies (such as explosions) exhibited more pronounced improvements. Even for anomaly types that were not explicitly synthesized diverse synthetic samples from other classes helped improve the model’s understanding of anomaly patterns, leading to consistent gains.

**Figure 6:**
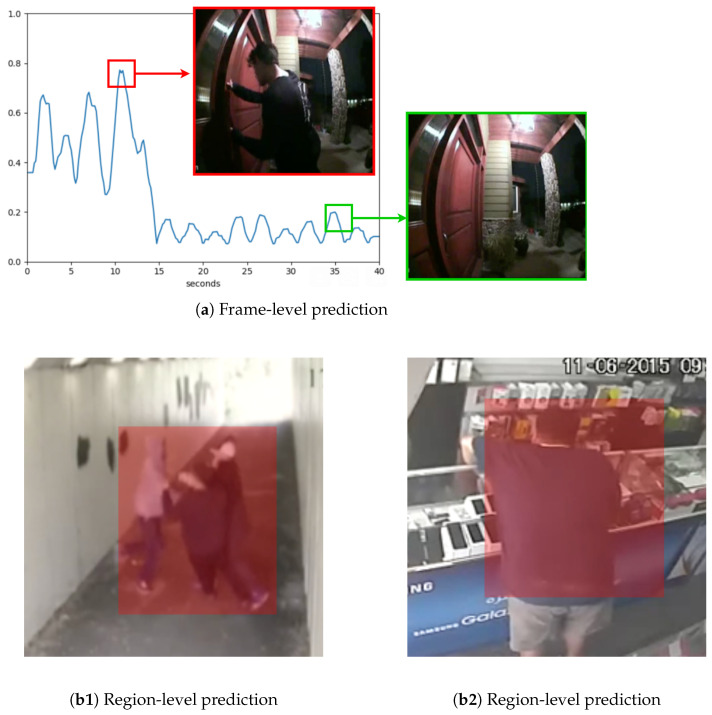
Visualization of frame-level and region-level predictions produced by our method.

Frame-Level Prediction. We evaluated the frame-level predictions of our method using 150 manually labeled samples drawn from both real UCF-Crime videos and our synthesized videos. To examine robustness under different evaluation regimes, we report the results on two test settings: a synthetic-only set and a mixed set containing both real and synthetic samples. Fine-grained anomaly detection was assessed across three semantic categories: Human–Human (e.g., an arrest or fighting), Human–Object (e.g., breaking or stealing), and No Human anomalies (e.g., fires and explosions). As shown in Table 6 and Figure 6, our model achieved strong performance across both test settings and all anomaly types, demonstrating its potential for practical security monitoring applications.

Furthermore, we conducted experiments to test the region-level predictions produced by our method. From Table 6, we can see that our method achieved promising accuracy in terms of region-level predictions. In addition, as shown in Figure 6, our predictions aligned well with the anomalous regions. It should be noted that these fine-grained evaluations were only used to analyze localization performance. All benchmark comparisons in Table 4 were conducted exclusively on the official UCF-Crime test set.

**Figure 7:**
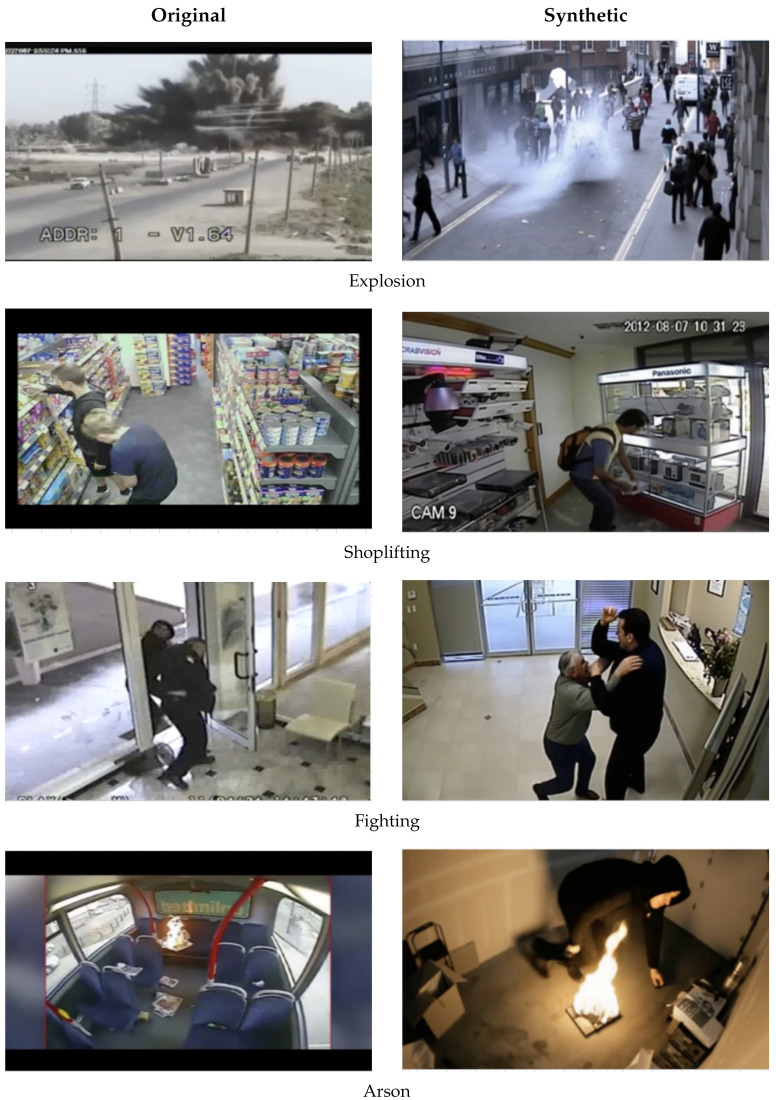
Examples of anomaly events from the UCF-Crime dataset (**left**) and our synthetic dataset (**right**).

Accuracy of Synthetic Annotations. The generated results are illustrated in Figure 7. To validate the accuracy of the fine-grained annotations produced by our generation framework, we further evaluated them using manually annotated labels as the ground truth. It can be observed from Table 6 that the synthetic annotations achieved an IoU of 0.76. This demonstrates the our synthetic fine-grained annotations are able to provide supervision with sufficient accuracy.

Realism of Synthetic Videos. To quantify the realism of the generated anomalous videos, we compared the Fréchet video distance (FVD) score on 20 synthetic videos for each anomaly type in Table 7. Our synthesized videos consistently achieved an average FVD of 98.23, indicating reasonable visual fidelity and temporal coherence with temporally consistent content.

### 4.4. Ablation Study

We further conducted ablation experiments to study the impact of prompt quality on the effectiveness of video synthesis.In our experiments, two prompt settings were employed:

- Basic Prompt: A short, minimal description of an anomaly type.
- Enriched Prompt: A detailed and structured description that included scene context, object interactions, and temporal cues.

Using the same framework, we trained models on videos generated with different prompt settings. Beyond visual quality, we also examined how these prompt types influenced the detection performance when used as additional training data. Specifically, we generated 100 synthetic anomaly videos using basic prompts and another 100 using enriched prompts. We used a model pretrained on existing VAD datasets to evaluate the generated videos and assess their quality. As shown in Table 8, the results show that the enriched prompts yielded higher video-level accuracy, indicating that higher-quality prompts led to more informative and reliable synthetic anomalies.

To further evaluate the effect of prompt quality on video generation itself, we conducted a semantic consistency check using GPT-based verification. For each video, 16 representative frames were sampled and compared with the original prompt for alignment evaluation. As summarized in Table 8, the enriched prompts produce videos with greater semantic consistency.

**Figure 8:**
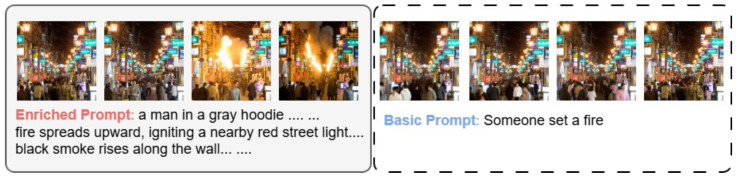
Visualization of fire event synthesized using basic vs. enriched textual prompts.

Figure 8 further shows that in complex scenes with crowded environments, specifying concrete actors and progressive actions enabled more accurate generation of fire-related anomalies. In contrast, simple prompts often failed to trigger the desired visual outcomes due to their lack of contextual grounding.

## 5. Conclusions

In this paper, we proposed an unsupervised anomaly video generation framework that synthesizes anomalous videos with fine-grained annotations from normal data. On top of this, we developed a baseline to achieve fine-grained video anomaly detection. Extensive experiments show that the generated data consistently improved video-level performance across multiple existing VAD methods. In addition, our baseline also produced accurate frame-level and region-level predictions, which demonstrates its effectiveness.

Following existing weakly supervised VAD settings and datasets, our detection framework predicted only one single anomalous bounding box per frame, which may not handle complex scenarios involving multiple anomalous events. Another limitation is that the generated videos still exhibited a domain gap compared with real-world surveillance videos, and some anomaly categories remained difficult to synthesize due to safety restrictions and generation failures. In addition, the high computational cost of our data generation pipeline hinders the production of a large-scale dataset. Moreover, frame-level and region-level evaluations were conducted on a relatively small set of manually annotated samples due to the lack of large-scale fine-grained benchmarks. In the future, we will extend our framework to support multiple anomaly detection, improve the efficiency of our data generation paradigm, and construct larger benchmarks for fine-grained video anomaly detection.

## Figures and Tables

**Table 1:** Comparison of existing VAD datasets in terms of annotation type, label density, scale, and scene diversity. The proposed dataset is highlighted in bold and ✓ indicates the presence of the corresponding property, whereas ✗ indicates its absence.

Dataset	Automatic Annotation	Dense Frame + Region Label	Video Count	Scene Diversity
**UCF-Crime [8]**	✗	✗	1900+	High
**UCF-Crime2Local [27]**	✗	✗	100 (subset)	High
**UCFCrime-Box [28]**	✗	✗	20 (subset)	Low
**UCF-Crime-DVS [29]**	✗	✗	1900+	High
**ShanghaiTech Campus [5]**	✗	✓	437	Low
**XD-Violence [9]**	✗	✗	4754	High
**Ours**	✓	✓	1012 (scalable)	High

**Table 2:** Comparison of existing datasets in terms of annotation type. The proposed dataset is highlighted in bold. ✓ and ✗ indicate whether the corresponding annotation is provided.

Dataset	Automatic Annotation	Fine-Grained Label
**UCF-Crime [8]**	✗	✗
**UCF-Crime2Local [27]**	✗	✗
**UCFCrime-Box [28]**	✗	✗
**UCF-Crime-DVS [29]**	✗	✗
**ShanghaiTech Campus [5]**	✗	✓
**XD-Violence [9]**	✗	✗
**Ours**	✓	✓

**Table 3:** Statistics of our synthetic videos. The total statistics are highlighted in bold.

Anomaly Type	Videos	Retention Rate (%)
Abuse	–	–
Arrest	8	30
Arson	80	94
Assault	36	58
Burglary	43	48
Explosion	70	97
Fighting	51	75
Road Accident	83	81
Robbery	35	52
Shooting	–	–
Shoplifting	78	64
Stealing	46	73
Vandalism	195	87
Normal	285	96
**Total**	**1012**	**79**

**Table 4:** Comparison of video-level anomaly detection performance on the UCF-Crime test set. Bold indicates results obtained by training with the proposed synthetic dataset.

Method	Training Data	AUROC (%)	Backbone
Sultani et al. [8]	UCF-Crime	75.41	C3D
RTFM [10]		84.03	I3D
WSAL [33]		85.38	I3D
S3R [34]		85.99	I3D
PEL [35]		86.76	I3D
MGFN [36]		86.98	VideoSwin
BN-WVAD [37]		86.88	I3D
Karim et al. [38]		86.94	I3D
STEAD-Fast [4]		88.87	X3D
**Sultani et al. [8]**	**UCF-Crime + Synthetic**	**78.23**	C3D
**BN-WVAD [37]**		**88.40**	I3D
**STEAD-Fast [4]**		**91.10**	X3D

**Table 5:** Video-level performance gains (%) brought about by synthetic data across anomaly categories.

Category	Arson	Explosion	Shooting	Vandalism
AUROC Gain (%)	+7.2	+8.2	+2.9	+1.3

**Table 6:** Fine-grained anomaly detection results on synthetic-only and mixed samples.

Anomaly Type	Frame AP (Syn/Mix) (%)	mIoU (Syn/Mix) (%)
Human–Human	0.74/0.79	0.64/0.67
Human–Object	0.57/0.50	0.59/0.65
No Human	0.65/0.53	0.68/0.73

**Table 7:** Fréchet video distance (FVD) between real and generated videos. For each anomaly class, FVD was computed using 20 real and 20 synthesized videos. Lower FVD values indicate greater realism. Bold indicates the average result, and ↓ denotes that lower values are better.

Anomaly Category	FVD ↓
Arson	72.50
Explosion	145.00
Fighting	112.40
Road Accident	84.10
Shoplifting	77.15
**Average**	**98.23**

**Table 8:** Semantic consistency results under different prompt settings. Retention rate was obtained from GPT-based verification, and model accuracy reflects the trained model’s ability to classify the generated videos.

Prompt Type	Synthetic Videos	Metric (%)
Basic Prompt	100	Retention: 40
		Model Acc.: 35
Enriched Prompt	100	Retention: 76
		Model Acc.: 89

## Data Availability

The public datasets analyzed in this study are available from their respective original sources. The synthetic dataset generated during this study will be made publicly available upon publication of this article.

## Supplementary Materials

The following supporting information can be downloaded at: https://www.mdpi.com/article/10.3390/s26134314/s1, Supplementary File S1: Code, Supplementary File S2: Showcase.MP4.
